# Awareness and use of ecological and economic evaluation of medical devices in healthcare centers: a survey of French hospitals

**DOI:** 10.1017/S0266462326104048

**Published:** 2026-08-04

**Authors:** Sophie Bouvet, Sarah Kabani, Manuel Roulaud, Elodie Benhaim, Thierry Chevallier, Virginie Chasseigne, Audrey Cordon, Sihame Chkair

**Affiliations:** 1 Department of Biostatistics, Epidemiology, Public Health, and Innovation in Methodology, Univ Montpellier, Centre Hospitalier Universitaire de Nîmes, France; 2 CHROME - Détection, évaluation, gestion des risques CHROniques et éMErgents, Nîmes Université, Nîmes, France; 3 PRISMATICS, INSERM, Centre d’investigation Clinique CIC 1402, University of Poitiers, France; 4 Service de Neurochirurgie du Rachis, Chirurgie de la Douleur & du Handicap (NRDH), Poitiers, CHU Poitiers, France; 5 Research and Innovation Department, CHU de La Réunion, France; 6 UMR 1302, Institute Desbrest of Epidemiology and Public Health, INSERM, Univ Montpellier, Montpellier, France; 7 Department of Pharmacy, Univ Montpellier, Centre Hospitalier Universitaire de Nîmes, France; 8 Clinical Epidemiology Unit (USMR), University Hospital Centre Bordeaux, France

**Keywords:** medical devices, procurement, economic evaluation, environmental impact, hospital-based HTA

## Abstract

**Objectives:**

The objective of this study is to examine current practices regarding the integration of environmental and economic impact assessments in decision making for medical device procurement across French healthcare facilities. These findings will reveal the barriers to environmental criteria integration in Hospital-Based Health Technology Assessment (HB-HTA).

**Methods:**

A cross-sectional survey, collected between 21 May and 12 July 2024, explored knowledge and implementation of environmental and economic assessments in hospital-based medical device procurement. Disseminated nationwide through the French Interregional Clinical Research and Innovation Groups (GIRCI), the French National Agency for Support to Healthcare Performance (ANAP) network, and via Territorial Hospital Groups, targeting public and private healthcare centers, the eight-section survey addressed governance, eco-responsible procurement, environmental data accessibility, and experience with joint assessments and certification practices.

**Results:**

Twenty-nine healthcare centers were analyzed, mostly regional or university hospitals or community hospitals (88 percent), primarily in the public sector. A structured approach to ecological transition was reported by 76 percent of institutions. Eco-responsible purchasing measures were implemented in nineteen centers (83 percent); however, access to life-cycle assessment data, especially for upstream manufacturing phases, remained limited. Forty-five percent of institutions had conducted joint environmental-economic assessments. Although 70 percent of respondents were aware of responsible procurement certifications schemes, only 36 percent used them.

**Conclusions:**

French healthcare institutes demonstrate growing awareness and engagement in sustainable procurement practices. However, limited access to standardized environmental data blocks systematic integration of environmental criteria into procurement practices and thus HB-HTA. Strengthening supplier transparency, harmonizing evaluation frameworks, and embedding environmental metrics into both processes could accelerate sustainable transition.

## Introduction

Sustainable development is a major societal priority. However, healthcare facilities have often been considered beyond the scope of environmental initiatives, considering that health outcomes outweigh environmental considerations (1). Healthcare delivery systems substantially contribute to environmental degradation and climate change (2). In France, the healthcare system represents ~75 million m^2^ of buildings, 15 percent of the energy consumption of the service sector, an average of 470 liters of water per hospital bed per day, and an estimated 8 percent of the national carbon footprint (3). Notably, around half of these greenhouse gas emissions are attributable to procurement, including medications (29 percent) and medical devices (21 percent) (3). Similar patterns occur internationally, with supply chain emissions accounting for the majority of the healthcare sector’s carbon footprint in Europe and globally (79 percent on average across OECD countries in 2018) (4).

In this context, sustainable development strategies have been gaining ground in institutional governance, including in healthcare facilities. The goal is to reconcile high-quality patient care by implementing a sustainable and economically viable healthcare system, while maintaining good working conditions for healthcare professionals.

A key lever to addressing healthcare emissions is health products procurement, namely pharmaceuticals and medical devices, which accounts for 50 percent of greenhouse gas emissions in the sector in France (3), while the supply chain represents 62 percent of the carbon footprint of the National Health Service in England (5). The medical device market is particularly relevant, being more exposed to competition, potentially facilitating environmentally informed purchasing decisions compared with medications or active molecules (6).

Hospital procurement is a legally guided process aiming to ensure a continuous, cost-effective supply of services. The recently added article 35 of the Climate and Resilience Act mandates environmental considerations in all public procurement contracts. Consequently, characterizing the drivers and barriers to integrating environmental criteria into hospital procurement is key to ensuring that these constraints are also appraised in hospital-based health technology assessments (HB-HTA). HB-HTA are commonly performed for expensive healthcare products, considering cost, clinical effectiveness, environmental impact, and broader institutional and organizational dimensions (7). A previous national survey reported that most hospitals used a formalized approach to HB-HTA, commonly considering economic data and device-specific clinical data, but fewer considered patient-related outcomes or post-marketing vigilance (8). However, this survey did not specifically question environmental considerations. Integrating environmental data into both procurement and HB-HTA processes may encounter common challenges, and determining the current application of combined environmental and economic assessments of medical devices used in healthcare facilities will optimize both green procurement and HB-HTA.

This study examines awareness, governance structures, implementation practices, and difficulties related to environmental and economic evaluation of medical devices. Our survey aimed to identify the facilitators and barriers to conducting procurement incorporating an environmental criterion in French healthcare facilities (9). These results may reveal common barriers to including environmental criteria in HB-HTA.

## Methods

### Study design

This study was an exploratory cross-sectional survey conducted by the GIRCI-SOHO (Regional Groups for Innovation and Cooperation – South West and Overseas territories; mainland administrative regions of Occitanie and Nouvelle-Aquitaine, plus La Réunion, Guadeloupe, Martinique, and French Guiana) working group on medical devices. The goal was to explore the contribution of economic and ecological studies to the evaluation of medical devices in hospitals.

The questionnaire comprised eight sections: (1) Institute structure characteristics; (2) Governance and strategic orientation; (3) Environmentally responsible purchasing; (4) Availability of environmental data; (5) Experience with joint environmental-economic assessments; (6) Responsible Supplier Relations and Sustainable Procurement Certification (RFAR); (7) Free comment section; and (8) Respondent profile. All questions were optional.

### Study preparation, administration, and data collection

The decision to run the survey was unanimously approved by the working group. A draft survey was developed, presented via video conference, and pretested internally by working group members (SB, MR, EB, AC, and SC). The pretest assessed question clarity and comprehension, feasibility and accessibility, questionnaire usability, and adequacy of topic coverage, leading to minor revisions. The survey was administered electronically and hosted on RedCap (10,11). The final version of the questionnaire is provided in Supplementary Appendix 1.

It was disseminated to French medical centers and via sustainable development expert networks. The questionnaire was sent to operational focal points and to the contact persons identified on the websites of each GIRCI. This represented a list of 189 initial contacts, and respondents were asked to forward the survey within their Territorial Hospital Group (GHT), including both public and private healthcare centers.

The questionnaire was sent to all GIRCI in France from 21 May to 12 July 2024, then further disseminated through the sustainable development experts within the ANAP (National Agency for the Support of Healthcare Performance) network on 13 June 2024.

Two reminder emails were sent to GIRCI members on 17 and 28 June. In addition, three university hospitals (CHUs) within the GIRCI were contacted by telephone to ensure comprehensive coverage of GIRCI-SOHO affiliated CHUs. The survey closed on 12 July 2024. Eligible respondents included hospital professionals directly involved in, or collaborating with, institutional committees for procurement, HTA, sustainable development, or institutional governance. Participants could report their job title in Section “Survey information.” As the questionnaire covered a wide range of expertise, it was expected that multiple respondents from the same institution would need to participate.

Each response was recorded as a single entry, corresponding to one row in the database. For centers that submitted multiple responses, manual data management was performed to reconstruct a single institutional answer, as the healthcare facility was our unit of analysis. This was operationalized as follows: (1) When a value was missing in one response but present in another, the available data were merged; (2) In cases of inconsistencies, we reconciled the data. Specifically, for difficulty ratings assessed using a visual analogue scale (VAS), the mean of the responses was retained. For the number of individuals involved in governance, the highest reported value was kept, assuming the other respondent had incomplete information regarding the full governance structure. Furthermore, as structural questions were optional, respondents from fully or partially anonymous centers could provide establishment details (e.g., sector of activity or the number of beds); conversely, named centers could choose not to answer these questions.

### Sample characteristics

The target sample included forty-eight national healthcare facilities affiliated with the seven GIRCI networks in France. To enhance representativeness, the targeted facilities were encouraged to distribute the survey within their GHTs. To further expand the sample, the network of sustainable development experts from ANAP was contacted.

Due to indirect dissemination, anonymization procedures, and redistribution within ANAP networks, it was impossible to determine the total number of institutions reached, preventing calculation of the participation rate. However, the representativeness of the sample relative to French healthcare facilities was assessed based on the diversity of the institutions’ profiles, structural characteristics, and location.

### Ethical considerations

This was an anonymous survey and no personal identifying information was collected. Although respondents could optionally indicate the name of the center, this information was neither reported nor analyzed. Data were analyzed by type of institution and by geographic region at the most detailed level. According to French regulations for anonymous survey research, institutional review board approval was waived.

### Statistical variables

Survey responses are presented descriptively. Continuous variables are presented as medians with interquartile ranges (IQR) and categorical variables as counts and percentages. Missing data were reported where applicable.

Considering the limited number of participating centers and the exploratory nature of the study, analyses were restricted to descriptive statistics. Inferential comparisons between regions or institution types were not performed. Data were analyzed using R software version 4.4.1 (R Core Team, 2024). A national participation map and corresponding graphs of the descriptive statistics were also generated using R.

## Results

### Respondent characteristics

A total of 112 clicks on the survey platform were recorded, many not leading to completed responses (opened to view only); eight centers submitted two responses, one center submitted four, and twenty centers submitted one. A simple row merge was sufficient for twenty-seven centers, and a data reconciliation process addressed inconsistencies; data were consolidated, resulting in twenty-nine responses considered as twenty-nine individual centers.

**Figure 1.**
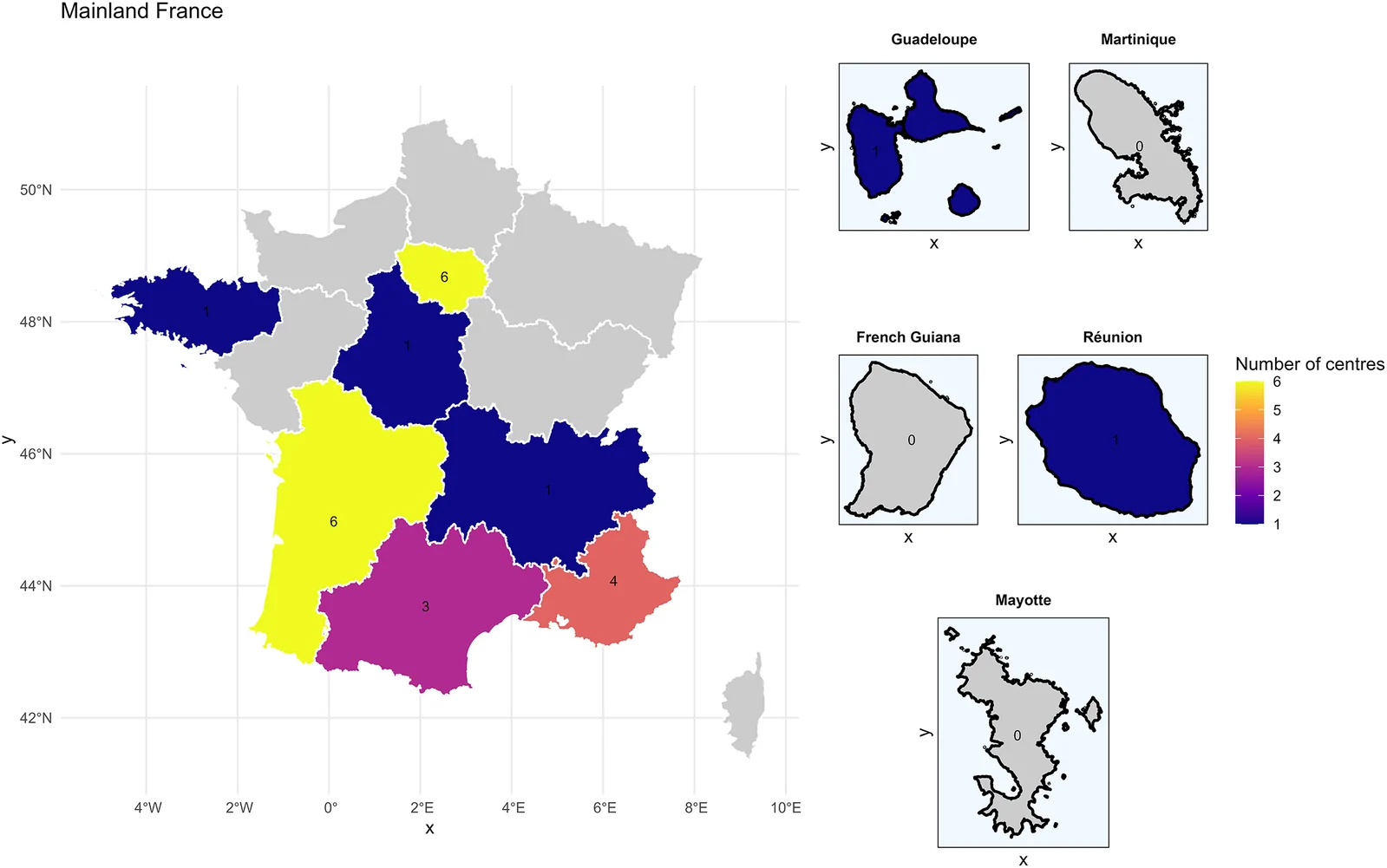
National Participation Map: Number of Participating Centers per Region in France. Figure 1. long description.

Of these, nineteen were identified by center name, six by region only, and seven were completely anonymous (i.e., nonreported center name or region). Most providing information on institute structure were regional or university hospitals (*n* = 13, 52 percent) or community hospitals (*n* = 9, 36 percent), predominantly public sector (*n* = 20, 91 percent), with a capacity of <2,000 beds (*n* = 17, 71 percent). No institutes provided information from five French regions and three overseas departments, with most responses from the South-west and Paris regions (*n* = 6 each) (Table 1). Two responses came from institutes in overseas territories. Nearly one quarter (11/48) of the GIRCI-SOHO hospital centers responded to the survey (Figure 1). Two cancer research centers completed only the identification section (center name and region) and so were excluded from the analyses.

**Table 1.** Results of the survey Table 1. long description.

Variable	Number (%) or median [IQR]
**Characteristic of centers**	**29**
Type of institute, *n* (%)	
Community hospital (CH)	9 (36)
University hospital (CHU) or Regional hospital (CHR)	13 (52)
Long-term care institute	1 (4.0)
Other	2 (8.0)
Missing	4
Sector, *n* (%)	
Private nonprofit clinic	2 (9.1)
Public establishment	20 (91)
Missing	7
Capacity – Number of beds, *n* (%)	
< 200	1 (4.2)
200–500	1 (4.2)
500–1000	6 (25)
1000–2000	9 (38)
2000–3000	6 (25)
3000–5000	1 (4.2)
Missing	5
Region, *n* (%)	
Auvergne-Rhône-Alpes	1 (4.2)
Bretagne	1 (4.2)
Centre-Val de Loire	1 (4.2)
Guadeloupe	1 (4.2)
Île-de-France	6 (25)
La Réunion	1 (4.2)
Nouvelle-Aquitaine	6 (25)
Occitanie	3 (13)
Provence-Alpes-Côte d’Azur	4 (17)
Missing	5
**Section 1**	**29**
Is there a management department, service, team, or committee dedicated to energy and ecological transition in healthcare within your organization?, *n* (%)	
Yes	22 (76)
Missing	0
Type of structuration, *n* (%) Management department Internal committee Specific service Unit or Team Other Missing	9 (40.9) 10 (45.5) 7 (31.8) 6 (27.3) 3 (13.6) 0
Number personnel, median [IQR]; missing Management department Internal committee Specific service Team	2 [0.5; 3]; 2 13.5 [7; 15]; 4 2 [2;2]; 4 1 [0.5; 2]; 3
**Section 2** Can you tell us about the overall actions and any guidelines implemented in your healthcare facility related to the ecological transition in healthcare? Structuring/governance of the energy and ecological transition in healthcare in your establishment Eco-design working groups for healthcare, made up of healthcare professionals from your institution Research projects in energy and ecological transition in health/environmental health promoted or initiated by your institution Sustainable Development/Corporate Social Responsibility/Energy and Ecological Transition Ambassadors in Healthcare within your institution Greenhouse gas emissions assessment including scope 3 carried out or planned for 2024 by your establishment Other actions carried out or planned by your institution Missing	**29** 19 (76); 4 15 (63); 5 11 (46); 5 18 (75); 5 13 (59); 7 16 (73); 7
**Section 3** In this context of ecological transition in healthcare, more specifically regarding the purchase of medical devices, are there any eco-responsible purchasing measures in place in your establishment? Yes Missing	**29** 19 (83) 6
**Section 4** The introduction of a medical device into a healthcare facility can result from a purchase contract, negotiations between the supplier and the facility (market access approach), or research. In these three identified contexts, which data do your institutions have (or request) access to? *N* (%) of Always, sometimes, never available; missing Case 1: Medical device purchased through a central purchasing agency Raw material extraction Manufacturing Transport Use for reusable medical devices End of life Case 2: Medical device purchased following direct negotiations with the supplier Raw material extraction Manufacturing Transport Use for reusable medical devices End of life Case 3: Medical device evaluated in a research context Raw material extraction Manufacturing Transport Use for reusable medical devices End of life	**29** 3 (23), 1 (8), 9 (69); 16 2 (17), 4 (33), 6 (50); 17 1 (8), 4 (33), 7 (58); 17 2 (17), 5 (42), 5 (42); 17 3 (25), 3 (25), 6 (50); 17 0, 4 (40), 6 (60); 19 0, 7 (70), 3 (30); 19 1 (10), 6 (60), 3 (30); 19 2 (20), 7 (70), 1 (10); 19 1 (9), 6 (55), 4 (36); 18 0, 2 (40), 3 (60); 24 0, 3 (60), 2 (40); 24 0, 2 (33), 4 (67); 23 0, 3 (50), 3 (50); 23 1 (17), 1 (17), 4 (67); 23
**Section 5** Have you ever conducted joint environmental plus economic assessments of medical devices?, *n* (%) Yes Missing	**29** 9 (45) 9
**If Yes,** On a scale of 0–10 (0 = Extremely difficult; 10 = Extremely easy), how would you rate it for the: Overall conduct of this assessment Qualified methodological resource to identify the list of data to be collected Collection of economic data Collection of environmental data Analysis and interpretation of the economic results Analysis and interpretation of the environmental results Median [IQR]; missing Did the results help inform the decision and change practices?, *n* (%) Yes Missing	9 4.5 [3;6]; 1 4.5 [2.5;5]; 1 8 [7.5;8.25]; 1 2.5 [2;3.5]; 1 7 [3.5;8.5]; 1 4 [2.5;5]; 1 5 (63) 1
**If No,** for what reason(s), *n* (%); missing Integrating an environmental criterion into medical device practices is not a priority for the institution. You have not received an official request for this. You do not have the human resources to carry out this type of assessment. You do not have the “expert” skills in this area. You do not have the financial resources to outsource this type of service. Other	11 (55) 2 (18); 0 1 (9); 0 7 (64); 0 5 (45); 0 5 (45); 0 3 (27); 0
**Section 6** Have you ever heard of Supplier Relations and Responsible Purchasing Charter certification [RFAR]?, n(%) Yes Missing	**29** 14 (70) 9
If Yes, does your medical device purchasing policy comply with RFAR certification (in whole or in part)? Yes Missing	14 4 (36) 3

Given the nature of the questions, no responses are presented in the table for sections 7 and 8.

The completion rates vary across domains: 90 percent; Section “Environmentally responsible purchasing”: 79 percent; Section “Availability of environmental data”: 48 percent; Section “Experience of joint environmental-economic assessments”: 69 percent; Section “Certification”: 69 percent; Section “Free comment section”: 66 percent; and Section “Survey information”: with 13/29 (45percent) centers completing all eight sections.

### Main findings

All results are summarized in Table 1.

#### Section 1: Institute structure

Most respondents (*n* = 22, 76 percent) reported the existence of a structured approach dedicated to global energy and environmental transition. This was commonly an internal committee (*n* = 10, 45.5 percent), a dedicated management department (*n* = 9, 40.9 percent), or a specific service (*n* = 7, 31.8 percent). In nine cases (40.9 percent), institutions relied on multiple levels of structuring (e.g., a committee combined with a management department). The terminology describing these structures was heterogeneous; although frequently used, the terms “sustainable development,” followed by “ecological transition” and “corporate social responsibility (CSR).”

The organizational modalities supporting environmental transition in healthcare differed both in terms of the type of structure and human resources. Committees involved the largest workforce, with a median of 13.5 [7; 15] dedicated staff members, compared with 2 for management departments [0.5; 3] and services [2; 2], and 1 in smaller teams [0.5; 2].

#### Section 2: Governance

The overarching actions and strategic orientations implemented by institutions primarily focused on structuring and governance (*n* = 19, 76 percent) and on sustainable development ambassadors’ networks (*n* = 18, 75 percent). Many institutions had adopted an organization based on working groups involving healthcare professionals dedicated to the eco-design of care processes (*n* = 15, 63 percent). Fifty-nine percent (*n* = 13) of respondents had completed or planned to conduct a greenhouse gas emissions assessment covering all upstream and downstream indirect emissions (Scope 3) for 2024.

Eleven centers (45.8 percent) reported research-related activity. Research topics included vulnerabilities of healthcare facilities to climate change, engagement of healthcare professionals in adaptation strategies and mitigation efforts, air pollution, life cycle assessments (LCA) (e.g., single-use versus reusable, care pathways), environmental impact of sterilization, occupational exposure, or during key life periods (pregnancy, first months of life, etc.). Some centers reported multiple ongoing projects and applications for internal and national calls for offers, or collaborations with CSR companies.

#### Section 3: Environmentally responsible purchasing

Nineteen centers (83 percent) reported having implemented eco-responsible purchasing measures. Two centers mentioned applying a 5 percent or 10 percent weighting to medical device tenders. Two institutes reported packaging optimization; others reported a plan to reduce single-use plastics or Di(2-ethylhexyl) phthalate and phthalate-free materials, or seeking new suppliers for outdated kits. One barrier raised was the limiting factor of medical devices being purchased via central purchasing groups. One mentioned SPASER (Scheme for the Promotion of Socially and Environmentally Responsible Public Procurement) implementation.

#### Section 4: Availability of environmental data

The availability of data to institutes when purchasing medical devices was tested for three situations: (1) Those purchased through central purchasing; (2) Those purchased following direct negotiation; and (3) Those used in a research context. Depending on the three situations, data availability was assessed as “always,” “sometimes,” or “never” for the phases corresponding to the LCA of medical devices: raw material extraction, manufacturing, transportation, use, and end of life.

**Figure 2.**
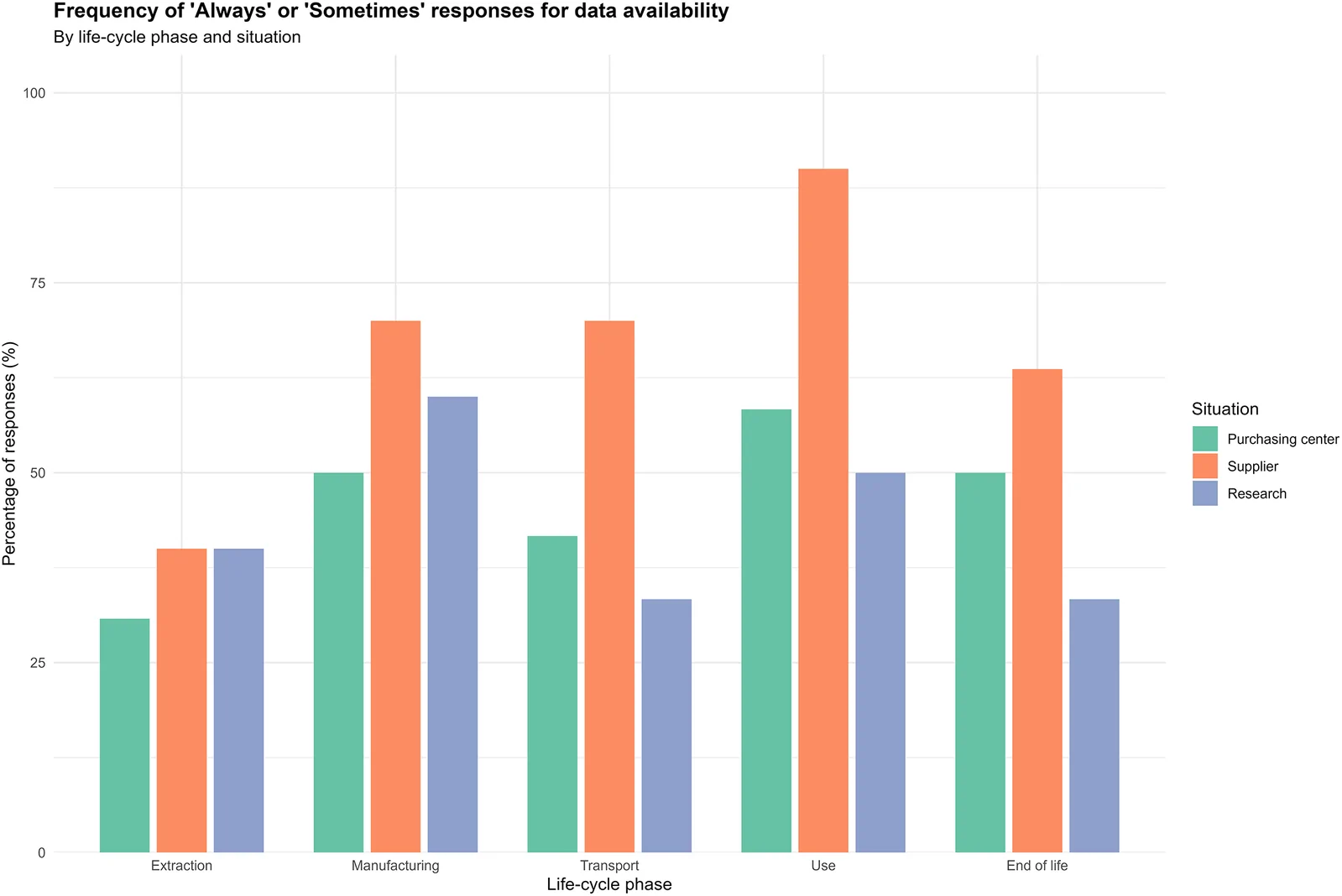
Data availability for environmental assessment depending on life-cycle phase and purchasing situation. Figure 2. long description.

Environmental information was most often available when purchasing directly from a supplier, regardless of the phase. Data availability was highest for the “use” phase, especially when purchased directly from manufacturers (90 percent), and lowest for the upstream phase of raw material extraction (31–40 percent) (Figure 2).

#### Section 5: Experience of joint environmental-economic assessments

**Figure 3.**
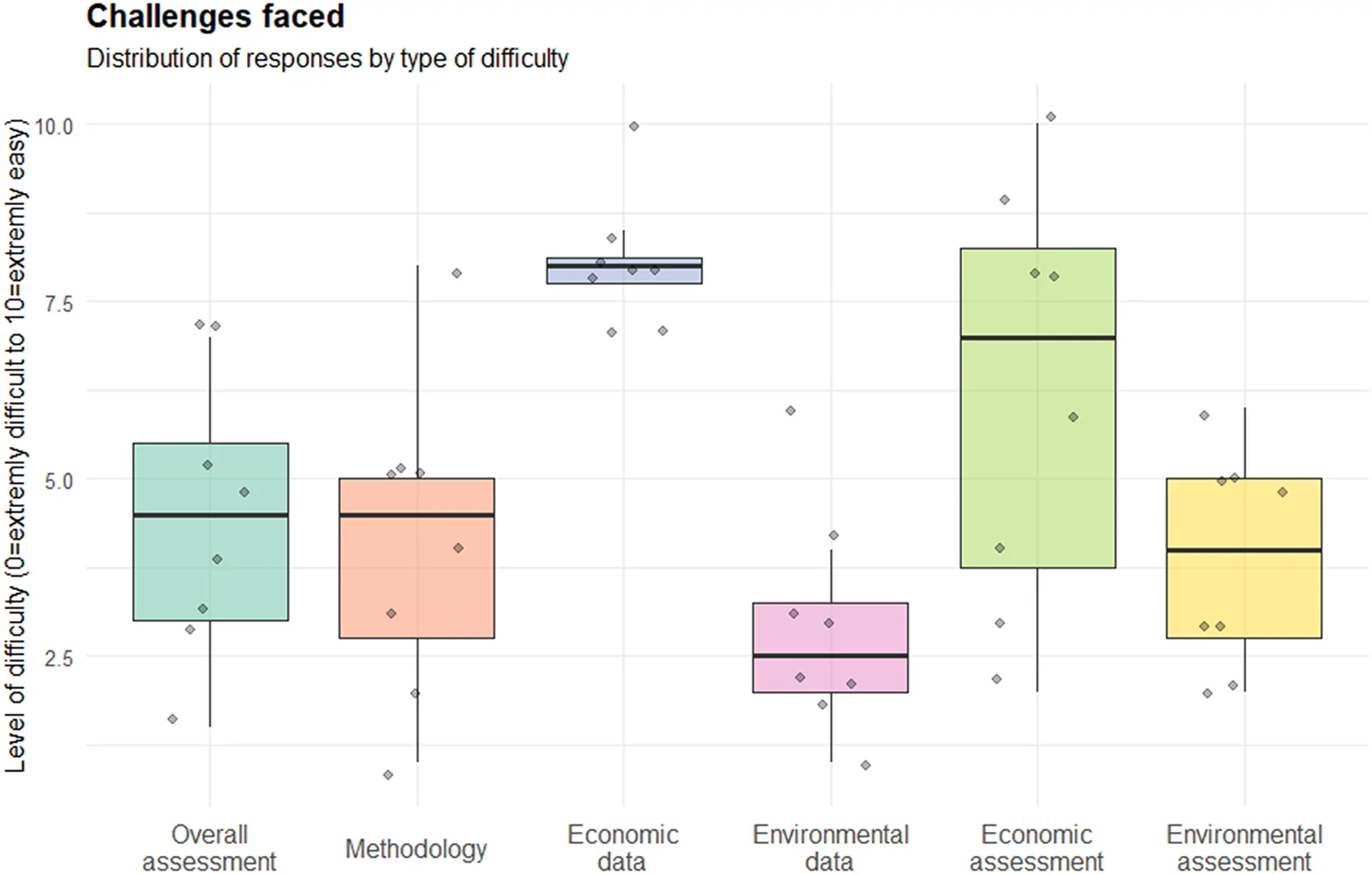
Challenges faced when performing a joint environmental and economic assessment. Figure 3. long description.

Joint environmental and economic assessments of medical devices were conducted in nine centers (45 percent). The main challenge was the collection of environmental data, followed by data analysis, with median difficulty scores of 2.50 [2.00, 3.50] and 4.00 [2.50, 5.00], respectively, on a scale from 0 (extremely difficult) to 10 (extremely easy) (Figure 3). Among centers performing such assessments, results informed decision-making and led to changes in practice in five centers (63 percent).

Among the eleven centers never conducting joint assessments, the main barriers were personnel requirements (*n* = 7, 63.6 percent), followed by need for specific expertise (*n* = 5, 45.5 percent) and financial resource requirements (*n* = 5, 45.5 percent). Less frequently cited barriers included lack of institutional priority (*n* = 2, 18.2 percent) and absence of an official request (*n* = 1, 9.1 percent). Nevertheless, seven (78 percent) indicated that conducting this type of assessment was under consideration.

#### Section 6: Certification

Fourteen (70 percent) of the centers had participants aware of the RFAR certification, of whom only four (36 percent) indicated that RFAR certification was taken into account in medical devices procurement decisions.

#### Section 7: Free comment section

Four respondents provided feedback on barriers to combined environmental and economic assessment of medical devices. Limited access to supplier data was a major obstacle, citing: “Easier access to supplier data and simplification/standardization of calculation methods for environmental/economic impact. Need for a national score. Difficult access, especially for upstream manufacturing data/raw material origin.”

One respondent highlighted the impact of transport and logistics optimization: “Some products may be ordered annually, and deliveries must follow a specific schedule to optimize transportation, for example.”

Another center emphasized difficulties in making multi-criteria decisions: “Environmental and economic requirements often remain contradictory in the short term. The supply of eco-responsible medical devices remains limited. Currently, the focus is primarily on identifying actual needs. In the future, it will be necessary to primarily question the relevance of disposable products and promote a return to reusable ones.”

Finally, two respondents desired standardized methodologies: “Simplified and standardized access to supplier data” and “Harmonized and shared methodology and tools.”

#### Section 8: Participant information

Participants had diverse professional backgrounds, reflecting the interdisciplinary nature of environmental decision making in healthcare institutions. Frequently, the questionnaire was completed by several individuals. Pharmacists contributed substantially (50 percent of respondents), along with sustainability or transition engineers (38 percent). Other respondents included stakeholders from material resources departments, CSR or environmental transition units, and senior management.

## Discussion

This national exploratory survey of environmentally aware purchasing within French healthcare facilities revealed a willingness to contribute, with numerous initiatives reported. Such initiatives, often driven by green-root groups or individuals, are increasingly being communicated, either through scientific publications (12–14) or via the ANAP and French Agency for Ecological Transition and Energy Management (ADEME) networks (15–17). Altogether, these contribute to an increasingly consolidated structuring of ecological transition in healthcare in France. Promisingly, 76 percent of centers had already implemented a structured approach to healthcare ecological transition.

This momentum is supported by direct integration of sustainability into institutional projects (18) and the national roadmap for ecological planning of the healthcare system (19) (Table 2). Furthermore, the French National Authority for Health (HAS) has introduced environmental criteria into the certification of healthcare institutions (20). In addition, the obligation to assess greenhouse gas emissions (BEGES) includes healthcare facilities, and a government decree (21) now requires the inclusion of Scope 3 for large public healthcare institutions. However, in our survey, only 59.1 percent of respondents had completed or planned to conduct a greenhouse gas emissions assessment including Scope 3 for 2024. Ignoring Scope 3 activities will hugely underestimate an institute’s footprint, as Scope 3 accounts for 50–75 percent of carbon emissions of healthcare settings (22) .

**Table 2.** Incentives and tools developed to encourage healthcare institutes to implement sustainable procurement practices Table 2. long description.

Sustainability initiatives/tools	Issuing body, country	Aims and scope
Article 35 of the Climate and Resilience Act	National Assembly, France	Obligatory environmental considerations in all public procurement contracts
Public Health Code. Article L6143–2 (2021)	National Assembly, France	The institute’s plan must include an eco-responsible component that defines objectives and a trajectory to reduce its carbon footprint
Environmental Health Roadmap (2023)	HAS, France	Decarbonizing the French healthcare system through 8 priority areas: buildings and energy, sustainable procurement, healthcare products, eco-responsible care, waste management, transport, digital health, and education and research
Certification of healthcare facilities for the quality of care	HAS, France	Introduction of environmental criteria into the certification of healthcare institutions
Greenhouse Gas Emissions Report (BEGES)	Government decree, July 2022, France	Obligation for large companies to conduct a greenhouse gas emissions assessment, including scope 3
Supplier Relations and Responsible Procurement Charter	National Responsible Procurement Pathway (PNAR), Ministry of the Economy, France	Signatories commit to 10 fundamental principles ensuring responsible financial relations with suppliers, considering life-cycle costs and impacts, and integrating environmental and social responsibility factors
RFAR	National Responsible Procurement Pathway (PNAR), Ministry of the Economy, France	Label that recognizes organizations demonstrating advanced maturity in responsible procurement management. RFAR has over 2300 signatories since launching in 2010
SPASER	France	Inclusion of social and environmental criteria in public hospital procurement contracts. This scheme is now mandatory for all public purchasers with an annual expenditure exceeding €100 million
National HTA frameworks	HAS, France; CADTH, Canada; NICE, UK	Roadmaps and guidelines to encourage committees to ask manufacturers to disclose their environmental data
Sustainable Medical Device Index (Index DM Durable)	C2DS and SNITEM, France	Enable buyers to incorporate CSR criteria into public and private procurement processes. Launched in June 2025
Euro-Pharmat recommendations for eco-responsible purchases	French Pharmaceutical Society of Medical Devices (SPFDM), France	Recommendations for sustainable procurement, providing practical tools to bridge the gap between technical expertise and environmental responsibility

BEGES: Bilan des Emissions de Gaz à Effet de Serre; CADTH: Canadian Agency for Drugs and Technologies in Health; C2DS: Committee for Sustainable Development in Healthcare; GHG: Greenhouse gas; HAS: French National Authority for Health; NICE: National Institute for Health and Care Excellence; SNITEM: National Union of the Medical Technology Industry; SPASER: Scheme for the Promotion of Socially and Environmentally Responsible Public Procurement: SPFDM: Société Pharmaceutique Française des Dispositifs Médicaux

The French government promotes responsible procurement through the National Responsible Procurement Pathway (PNAR), an initiative to improve relationships between buyers and suppliers and embed CSR principles within the procurement functions. This pathway comprises the Supplier Relations and Responsible Procurement Charter and the Supplier Relations and Responsible Procurement Label (RFAR) (Table 2). Nevertheless, only 36 percent of facilities in our survey considered RFAR certification when purchasing medical devices. Another policy instrument, SPASER, recently adopted by the national UniHA purchasing group, requires social and environmental criteria in public hospital procurement contracts (Table 2). Such structural initiatives align centralized purchasing mechanisms with national sustainability objectives.

In Finland, a survey of district hospitals found that 54 percent (7/13) of institutes considered sustainability in their procurement process, with a heterogeneous list of measures considered: packaging, life-cycle costing, availability of spare parts, disposal and so forth (23). Besides, 25 percent of the responding organizations used a voluntary GPP guide within their procurement decision making (23). In France, our findings that 45 percent of centers have already carried out a joint environmental and economic assessment of medical devices reflect similar local initiatives; these are generally conducted before the purchase or tender process to guide selection between products.

The French healthcare sector accounts for 8 percent of the national carbon footprint, 21 percent of which is attributable to the purchase of medical devices. The medical device market is highly competitive, which could favor healthcare institutions seeking to improve environmental credentials. Traditionally focused on cost, hospital purchasers may increasingly prioritize sustainable options, necessitating standardized comparative methods and access to data (24). Our survey revealed difficulties in accessing LCA data, in particular for raw material extraction, with challenges in analyzing these data (9). Inaccessibility of proprietary data supports previous studies encountering this limitation when comparing medical devices (25), and is a major barrier across the international HTA literature (26–28). Combined with a lack of time and resources, this scarcity requires an adaptation of practices to streamline hospital-level efforts through the concept of “triage” (26). Consequently, comprehensive environmental impact assessments should be prioritized for healthcare products with high-stakes ecological implications (e.g., transition from single-use plastics to reusable alternatives, or devices generating toxic waste), thereby ensuring maximum ecological impact for each analysis performed. While procurement and HB-HTA are distinct processes, they represent interconnected dimensions of technology selection, sharing data needs, multi-stakeholder committees, and decision-making criteria (9). The lack of standardized environmental data and methodological uncertainty delays integration of environmental sustainability into formal HTA frameworks. Indeed, an international survey of HTA organizations found a stark gap between high awareness and low implementation: while most organizations recognized the importance of environmental sustainability, only 35percent (9/26) actively embedded these considerations into HTA practices, citing data collection difficulties as roadblocks (24). This aligns with our findings, where hospital buyers struggle with raw data analysis.

To increase transparency in medical device procurement, the Sustainable Medical Device Index (29) enables buyers to incorporate CSR criteria into procurement processes. Other initiatives aim to bridge this gap at the European level (30). Suppliers looking to capture additional market share will likely prioritize sustainability, ethical standards, and transparency (31).

Such procurement-driven tools are valuable for HB-HTA, serving as a practical entry point to drive the data generation and methodological advancement required for broader technology assessments (9). Thus, according to Toolan’s four methodological approaches (27), the procurement process could encompass an “Information Conduit” method using these tools, whereas HB-HTA, a high-value, multidisciplinary process, could integrate more comprehensive methods such as “Parallel” or “Integrated” evaluation.

The diversity of survey participants highlights the need for structured communication between stakeholders. Pharmacists ensure appropriate use of medical devices, while purchasers focus on economic sustainability, sustainability or transition officers manage environmental impact (32), and senior management provide overall validation and strategic oversight. In addition, hospital clinicians and patients, as the end users of medical devices, must also be considered. Certainly, in discrete choice experiments, patients clearly prefer environmentally sustainable medications (33). Professional bodies such as Euro-Pharmat are developing recommendations for sustainable procurement, providing tools to bridge the gap between technical expertise and environmental responsibility (34).

This complex interplay of hospital stakeholders supports the HTA literature. Integrating environmental criteria requires interdisciplinary teams (e.g., environmental scientists and clinical and biomedical engineers) (24). Our survey demographics mirror this recommendation, with substantial contributions from pharmacists (50 percent) and sustainability or transition engineers (38 percent). However, we found that expertise within French hospitals is fragmented across different departments (e.g., material resources, CSR units, and senior management). Furthermore, Ardito et al. (9) noted that moving beyond a narrow healthcare payer perspective toward a broader societal perspective introduces complex trade-offs between immediate clinical/economic constraints and long-term environmental benefits. Navigating these ethical and practical trade-offs requires the exact structured collaboration among pharmacists, clinicians, and management that our survey identified.

Several centers reported that they drafted activity reports, policies, and action plans that are not systematically shared between institutes. A greater transparency and exchange of implementation policies would help advance such initiatives in late-adopting centers and those without dedicated resources.

National HTA frameworks are designing roadmaps that will encourage manufacturers to disclose environmental data, although these boards primarily focus on regulatory approval, pricing, and reimbursement (35–37). Conversely, local HB-HTA (e.g., COMEDIMS) take a pragmatic and context-dependent approach, arbitrating between competing medical devices, managing local waste constraints, and adapting to local CSR goals. Therefore, there is an urgent need for national agencies to provide standardized grids and evaluation toolkits for hospitals (28). Overall, the findings suggest that ecological transition in French healthcare is moving from a voluntary and fragmented phase toward a more regulated and structured model. Rather than viewing hospital procurement and HB-HTA as separate, siloed areas, they should be understood as a multilevel continuum. Establishing a culture of environmental accountability and data transparency in hospital procurement is a prerequisite for obtaining reliable data to conduct rigorous, multi-criteria HTA in hospitals.

### Limitations

Participating centers were located primarily in the South-west and around the capital, with no centers in the North or North-east. Three of the five overseas territories also did not respond. These geographical gaps limit the generalizability of our findings. Moreover, the sampling strategy relied on network-based dissemination and voluntary participation, introducing potential self-selection bias. The centers least invested in sustainability might have been less likely to respond. The total number of institutions reached could not be determined; therefore, the participation rate could not be calculated. Consequently, statistical representativeness could not be formally assessed. Furthermore, the data collection window was short due to an internal reporting deadline. Nevertheless, despite the modest sample size, this distribution includes key institutional profiles from the French public healthcare sector, which manages the majority of acute care beds and carries the heaviest procurement volumes. Therefore, these findings provide a valuable, exploratory cross-section of the primary buyers driving the ecological transition.

Some sections had high levels of missing data, due to the challenges in gathering information from different staff members within a single center. Indeed, only 48 percent responded to Section “Availability of environmental data.” This introduces a selection bias whereby responding centers likely represent the most advanced facilities. Consequently, findings regarding data availability and specific certifications should be interpreted as a “best-case scenario” driven by environmentally mature centers.

Moreover, all questions were optional, and some were open-ended, resulting in differing levels of information from centers. The questionnaire was internally pretested but did not undergo formal external validation. Overall, findings should be interpreted as exploratory and descriptive rather than generalizable across French healthcare institutions.

## Conclusion

This national survey shows that ecological transition is becoming entrenched throughout French healthcare institutions, with governance frameworks and concrete actions, particularly regarding eco-responsible procurement. However, inadequate access to environmental data, especially for upstream phases of the life cycle of medical devices, remains a major obstacle to establishing the reliable data foundations needed for future HB-HTA. Standardizing methods, increasing supplier transparency, and sharing resources between institutions will accelerate wider adoption of sustainable procurement practices. Institutions concerned about their environmental impact wield an incentive lever with suppliers seeking market share, which can promote the emergence of more sustainable products.

Although we focused on procurement practices rather than HTA frameworks, the data gaps and fragmentation of responsibilities identified suggest that the systematic integration of environmental criteria into HB-HTA remains a long-term prospect. Progress in green procurement provides a stepping stone toward this goal, ultimately enabling the assessment of the full life-cycle value and sustainability of medical technologies.

Finally, although regulations and certifications encourage action, their implementation remains uneven, highlighting the need for stronger support to assist institutions with less experience in the ecological transition.

## Supporting information

10.1017/S0266462326104048.sm001 Bouvet et al. supplementary material Bouvet et al. supplementary material

## Supplementary material

The supplementary material for this article can be found at http://doi.org/10.1017/S0266462326104048.

## Long descriptions

### Table 1. Long description

The table is divided into a header row and several thematic sections. The header columns are Variable and Number (percentage) or median [I Q R].

* Characteristic of centers (n = 29): Most are University or Regional hospitals (52 percent) or Community hospitals (36 percent). 91 percent are public establishments. The largest group by capacity has 1000 to 2000 beds (38 percent). Geographically, the highest concentrations are in Ile-de-France (25 percent) and Nouvelle-Aquitaine (25 percent).

* Section 1: 76 percent have a dedicated department for ecological transition. Structuring includes Internal committees (45.5 percent) and Management departments (40.9 percent). Median personnel counts range from 1 in teams to 13.5 in internal committees.

* Section 2: Overall actions include structuring/governance (76 percent), ambassadors (75 percent), and eco-design working groups (63 percent).

* Section 3: 83 percent have eco-responsible purchasing measures for medical devices.

* Section 4: Data availability for medical devices across three cases (Central agency, Direct negotiation, Research) and the life cycle assessement 5 steps. Data availability is highest in the use step and worst in the raw material extraction step.

* Section 5: 45 percent have conducted joint environmental and economic assessments. On a 0 to 10 scale, collecting economic data is rated easy (median 8), while collecting environmental data is difficult (median 2.5). For those who have not, the main reason is lack of human resources (64 percent).

* Section 6: 70 percent have heard of R F A R certification, and 36 percent of those comply with it.

Navigate back to Table 1..

### Figure 1. Long description

The map of France shows its regional divisions as well as its five overseas departments. The regions with the most centres are Île-de-France and Nouvelle-Aquitaine, with six centres each, followed by Provence-Alpes-Côte d’Azur with four centres and Occitanie with three centres. Finally, Brittany, Centre-Val de Loire and Auvergne-Rhône-Alpes are each represented by one centre. The remaining six regions, which had no respondents, are located in the northern half of France (Pays de la Loire, Normandy, Hauts-de-France, Grand Est and Bourgogne-Franche-Comté) or the far south (Corsica). Finally, of the five overseas territories, two responded to our survey: Guadeloupe and Réunion.

Navigate back to Figure 1..

### Figure 2. Long description

The chart compares the percentage of “Always or Sometimes” responses regarding environmental data availability across three scenarios (Purchasing centre, Supplier, Research) and five LCA phases: Extraction: Data availability is 31% for Purchasing centre, and 40% for both Supplier and Research. Manufacturing: Data availability rises to 50% for Purchasing centre, 60% for Research, and 70% for Supplier. Transport: Availability is lowest for Research (33%), followed by Purchasing centre (42%) and Supplier (70%). Use: Availability reaches 50% for Research, 59% for Purchasing centre, and peaks at 90% for Supplier. End of Life: Availability stands at 34% for Research, 50% for Purchasing centre, and 64% for Supplier. Key takeaway: Across all five LCA phases, data is consistently most available in the “Supplier” scenario.

Navigate back to Figure 2..

### Figure 3. Long description

On a scale of 0 to 10 (0 = Extremely difficult; 10 = Extremely easy), diffculty rating is assessed by median avec IQR:

-Overall conduct of this assessment: 4.5 [3;6]

-Qualified methodological resource to identify the list of data to be collected: 4.5 [2.5;5]

-Collection of economic data: 8 [7.5;8.25]

-Collection of environmental data: 2.5 [2;3.5]

-Analysis and interpretation of the economic results: 7 [3.5;8.5]

-Analysis and interpretation of the environmental results: 4 [2.5;5]

Navigate back to Figure 3..

### Table 2. Long description

Table summarizing 11 sustainability initiatives and tools.

Article 35 (Climate Act) — Nat. Assembly, FR. Mandatory environmental criteria in public procurement.

Public Health Code Art. L6143-2 — Nat. Assembly, FR. Eco-responsible plans to cut carbon footprints.

Environmental Health Roadmap — HAS, FR. Decarbonizes healthcare across 8 priority areas.

Healthcare Certification — HAS, FR. Integrates green criteria into facility certification.

BEGES Report — Decree, FR. Requires large companies to assess scope 3 emissions.

Responsible Procurement Charter — PNAR, FR. Principles for sustainable supplier relations.

RFAR — PNAR, FR. Label for advanced responsible procurement.

SPASER — FR. Mandatory ESG criteria for purchasers >€100M.

National HTA Frameworks — HAS, CADTH, NICE. Encourages environmental data disclosure.

Sustainable Medical Device Index — C2DS/SNITEM, FR. CSR criteria in procurement.

Euro-Pharmat Recommendations — SPFDM, FR. Practical tools for sustainable procurement.

Navigate back to Table 2..
