# Supplementary material for: Awareness and use of ecological and economic evaluation of medical devices in healthcare centers: a survey of French hospitals
Source: Int J Technol Assess Health Care. 2026 Aug 4;42(1):e82. doi: 10.1017/S0266462326104048 (PMC13735843; doi:10.1017/S0266462326104048)
Supplement: Supplementary file 1 [file S0266462326104048sup001.pdf]

Appendix 1 : Questionnaire

Characteristics of your institute:

Type of institute:

☐ University hospital (CHU) ☐ Regional hospital (CHR) ☐ Community hospital (CH) ☐ Private non-profit clinic ☐ Private for-profit clinic ☐ Public-Private establishment

Capacity – Number of beds:

☐ < 200 ☐ 200 to 500 ☐ 500 to 1000 ☐ 1000 to 2000 ☐ 2000 to 3000 ☐ 3000 to 5000 ☐ > 5000

Types of care:

☐ Acute care (medicine, surgery, obstetrics) ☐ Post-acute and rehabilitation care ☐ Long-term care ☐ Psychiatric ☐ Hospital-at-home ☐ Nursing home ☐ A&E ☐ Dialysis center

Region: [Scroll-down menu]

[Tapez ici]

Question 1:

Is there a department, service, unit, or team dedicated to the energy and ecological transition in healthcare within your organization? ☐ Yes/ ☐ No

If yes:

It is:	Number of people
☐ a management department,<br>please specify its name:	|__||__|
☐ a department / service,<br>please specify its name:	|__||__|
☐ a unit / team,<br>please specify name and domain:	|__||__|
☐ a committee within the establishment, please specify its name:	|__||__|
☐ Other, please specify:	|__||__|

Question 2: Can you tell us about the overall actions and any guidelines implemented in your healthcare facility related to the ecological transition in healthcare?

Structuring / governance of the energy and ecological transition in healthcare in your establishment

☐ No/ ☐ Yes

If yes, please describe:

____

____

Eco-design working groups for healthcare, made up of healthcare professionals from your institution ☐ No / ☐ Yes

If yes, please describe:

____

____

Research projects in energy and ecological transition in health / environmental health promoted or initiated by your institution ☐ No / ☐ Yes

If yes, please describe:

____

____

Sustainable Development / Corporate Social Responsibility / Energy and Ecological Transition Ambassadors in Healthcare within your institution ☐ No / ☐ Yes

If yes, please describe:

____

____

Greenhouse gas emissions assessment including scope 3 carried out or planned for 2024 by your establishment ☐ No / ☐ Yes

If yes, please describe:

____

____

Other actions carried out or planned by your institution: ☐ No / ☐ Yes

If yes, please describe:

____

____

Question 3: In this context of ecological transition in healthcare, more specifically regarding the purchase of medical devices, are there any eco-responsible purchasing measures in place in your establishment? ☐ No / ☐ Yes

If yes, please describe:

____

____

Question 4: The introduction of a medical device into a healthcare facility can result from a purchase contract, negotiations between the supplier and the facility (market access approach), or research.

In these three identified contexts, which data do your institutions have (or request) access to?

Case 1: Medical device purchased through a central purchasing agency:

Type of data	Always available	Sometimes available	Never available
Raw material extraction: detailed list of all components: nature and weight. Including packaging.			
Manufacturing: manufacturing processes (sterilization, energy consumption of the processes, country of manufacture).			
Transport: distances between the different stages (raw material extraction, manufacturing, packaging, shipping, distribution, etc.) and modes of transport used.			
Use for reusable medical devices: lifespan, associated consumption during operation (energy, chemicals, water, etc.), maintenance methods (sterilization, laundry, disinfection, etc.) with associated consumption.			
End of life: disposal channel for each medical device (household waste, infectious medical waste, recycling) with the locations of treatment facilities and the type of final recovery (incineration, landfill, material recovery).			

Case 2: Medical device purchased following direct negotiations with the supplier:

Type of data	Always available	Sometimes available	Never available
Raw material extraction: detailed list of all components: nature and weight. Including packaging.			
Manufacturing: manufacturing processes (sterilization, energy consumption of the processes, country of manufacture).			
Transport: distances between the different stages (raw material extraction, manufacturing, packaging, shipping, distribution, etc.) and modes of transport used.			
Use for reusable medical devices: lifespan, associated consumption during operation (energy, chemicals, water, etc.), maintenance methods (sterilization, laundry, disinfection, etc.) with associated consumption.			
End of life: disposal channel for each medical device (household waste, infectious medical waste, recycling) with the locations of treatment facilities and the type of final recovery (incineration, landfill, material recovery).			

Case 3: Medical device evaluated in a research context:

Type of data	Always available	Sometimes available	Never available
Raw material extraction: detailed list of all components: nature and weight. Including packaging.			
Manufacturing: manufacturing processes (sterilization, energy consumption of the processes, country of manufacture).			
Transport: distances between the different stages (raw material extraction, manufacturing, packaging, shipping, distribution, etc.) and modes of transport used.			
Use for reusable medical devices: lifespan, associated consumption during operation (energy, chemicals, water, etc.), maintenance methods (sterilization, laundry, disinfection, etc.) with associated consumption.			
End of life: disposal channel for each medical device (household waste, infectious medical waste, recycling) with the locations of treatment facilities and the type of final recovery (incineration, landfill, material recovery).			

Question 5: Have you ever conducted joint environmental **plus** economic assessments of medical devices?

☐ Yes/ ☐ No

If yes,

1/ On a scale of 0 to 10 (0 = Extremely difficult; 10 = Extremely easy), how would you rate it for:

-The overall conduct of this assessment:

- Access to a qualified methodological resource to identify the list of data to be collected:

- The collection of economic data:

- The collection of environmental data:

- The analysis and interpretation of the economic results:

- The analysis and interpretation of the environmental results:

2/ Did the results help inform the decision and change practices? ☐ Yes / ☐ No

If yes, can you provide one or more examples?

____
____

If no:

1/ For what reason(s):

☐ Integrating an environmental criterion into medical device practices is not a priority for the institution.

☐ You have not received an official request for this.

☐ You do not have the human resources to carry out this type of assessment.

☐ You do not have the "expert" skills in this area.

☐ You do not have the financial resources to outsource this type of service.

☐ Other, please specify:

2/ Is conducting joint environmental plus economic assessments of medical devices a prospect for your organization? Yes / No

If yes, what type of action is planned?

____
____

Question 6:

Have you ever heard of Supplier Relations and Responsible Purchasing Charter certification (**[RFAR])**?

☐ Yes / ☐ No

If yes, does your medical device purchasing policy comply with RFAR certification (in whole or in part)?

☐ Yes / ☐ No

Question 7: Do you have any specific suggestions/comments about the joint environmental plus economic assessment of medical devices?

____
____

Question 8:

Who participated in this survey? (multiple responses possible):

☐ General Management

☐ Corporate Social Responsibility / Energy and Ecological Transition Department

☐ Energy and Ecological Transition Engineer / Advisor in Healthcare

☐ Energy and Ecological Transition Technician in Healthcare

☐ Material Resources Department

☐ Other, please specify:
